# Effects of earplugs and eye masks combined with relaxing music on sleep, melatonin and cortisol levels in ICU patients: a randomized controlled trial

**DOI:** 10.1186/s13054-015-0855-3

**Published:** 2015-03-27

**Authors:** Rong-Fang Hu, Xiao-Ying Jiang, Kathleen M Hegadoren, You-Hua Zhang

**Affiliations:** School of Nursing, Fujian Medical University, 1 Xue Yuan Road, University Town, Fuzhou, 350108 China; Faculty of Nursing, University of Alberta, 11405 87 Avenue, Edmonton, Alberta T6G 1C9 Canada; Department of Nuclear Medicine, Fujian Province Hospital, East Street 134, Fuzhou, 350001 China

## Abstract

**Introduction:**

Intensive care unit (ICU) environmental factors such as noise and light have been cited as important causes of sleep deprivation in critically ill patients. Previous studies indicated that using earplugs and eye masks can improve REM sleep in healthy subjects in simulated ICU environment, and improve sleep quality in ICU patients. This study aimed to determine the effects of using earplugs and eye masks with relaxing background music on sleep, melatonin and cortisol levels in ICU patients.

**Methods:**

Fifty patients who underwent a scheduled cardiac surgery and were expected to stay at least 2 nights in Cardiac Surgical ICU (CSICU) were included. They were randomized to sleep with or without earplugs and eye masks combined with 30-minute relaxing music during the postoperative nights in CSICU. Urine was analyzed for nocturnal melatonin and cortisol levels. Subjective sleep quality was evaluated using the Chinese version of Richards-Campbell Sleep Questionnaire (a visual analog scale, ranging 0–100).

**Results:**

Data from 45 patients (20 in intervention group, 25 in control group) were analyzed. Significant differences were found between groups in depth of sleep, falling asleep, awakenings, falling asleep again after awakening and overall sleep quality (*P* < 0.05). Perceived sleep quality was better in the intervention group. No group differences were found in urinary melatonin levels and cortisol levels for the night before surgery, and the first and second nights post-surgery (*P* > 0.05). The urinary melatonin levels of the first and second postoperative nights were significantly lower than those of the night before surgery (*P* = 0.01). The opposite pattern was seen with urinary cortisol levels (*P* = 0.00).

**Conclusion:**

This combination of non-pharmacological interventions is useful for promoting sleep in ICU adult patients; however, any influence on nocturnal melatonin levels and cortisol level may have been masked by several factors such as the timing of surgery, medication use and individual differences. Larger scale studies would be needed to examine the potential influences of these factors on biological markers and intervention efficacy on sleep.

**Trial registration:**

Chinese Clinical Trial Registry: ChiCTR-IOR-14005511. Registered 21 November 2014.

## Introduction

Sleep is a basic need for human beings and is especially important for healing and survival in critical illness [1,2]. Sleep deprivation impairs immune function, decreases inspiratory muscle endurance, negatively affects weaning from mechanical ventilation, prolongs ICU stay and has been associated with delirium and mortality in the ICU [1,3-6]. Yet previous studies have been consistent in describing the poor sleep of ICU patients. A number of polysomnography (PSG) studies have shown that ICU patients commonly have broken, light sleep with a lack of slow-wave sleep and rapid eye movement (REM) sleep [6-9]. Meanwhile, surveys have identified poor sleep as one of the most frequent complaints among ICU survivors [5,10].

Numerous factors including sedation, environmental factors, disease and mechanical ventilation have been reported to contribute to sleep disturbance in ICU [5,11,12]. Evidence has suggested that excessive noise and continuous light exposure are common in ICU settings [4,8,13-15]. Noise has been widely cited as the most common cause of sleep disruption in the critically ill [14,16].

The World Health Organization (WHO) has recommended that the average noise levels in hospital wards should not exceed 30 dB (A) during day or night, and peak levels should not exceed 40 dB (A) during the night [17]. Unfortunately, most studies have shown that noise levels in the ICU are much higher than these recommendations. The peak noise levels in the ICU routinely exceed 80 dB (A) [4,8,13,14,16]. The equivalent sound pressure level exceeding 30 dB (A) indoors for continuous noise and peak noise levels at 45 dB (A) or less may negatively affect sleep and result in sleep disturbance [17]. More than 70 dB (A) of noise may result in vasoconstriction, increased heart rate, hypertension and even arrhythmias [18].

Moreover, continuous light exposure is another noxious and disruptive environmental factor affecting sleep in the ICU. Light plays a vital role in synchronization of the circadian rhythm. Chang *et al*. found that light levels of the range of approximately 30 to 50 lux in the angle of gaze delayed the circadian clock, acutely suppressed melatonin and disrupted sleep [19]. Chellappa *et al*. reported that light can impact directly upon sleep structure at low light levels (40 lux) [20]. Continuous light measurements made in four ICUs show that the mean maximum nocturnal level ranges from 128 to 1,445 lux, which is high enough to suppress melatonin, and may affect sleep and biological rhythm [4].

In the past 20 years, multiple strategies have been proposed to optimize sleep in the ICU. A number of studies have been carried out on the effects of non-pharmacologic interventions for sleep promotion in ICU patients [21-25]. Using protective devices such as earplugs and eye masks and listening to music are important options in this field [23-25], although no clinical studies have been published that used all three strategies in combination. Several studies have investigated applying protective earplugs and eye masks in ICU patients or in an ICU simulated environment [24-28]. In our previous study with healthy adults in a simulated ICU environment, using earplugs and eye masks improved REM sleep, reduced REM sleep latency and arousals indices, and affected nocturnal melatonin and cortisol secretion levels [28]. Our aim in this study was to evaluate the effects of using earplugs and eye masks, combined with listening to music, on sleep and hormone secretion in ICU patients.

We hypothesized that a reduction of noise and light during the night using earplugs and eye masks combined with listening to sleep-inducing music could be beneficial in sleep promotion and the protection of nocturnal melatonin and cortisol secretion in ICU patients. To test this hypothesis, a randomized controlled clinical trial (RCT) was conducted in cardiac surgical patients during postoperative nights in an ICU.

## Materials and methods

This study was a prospective single-center randomized controlled parallel-group clinical trial performed within a 21-bed Cardiac Surgical Intensive Care Unit (CSICU) of Fujian Medical University Union Hospital, Fuzhou, China. It was approved by the Hospital and Fujian Medical University Research Ethics Boards. The trial was registered in the Chinese Clinical Trials Registry (ChiCTR-IOR-14005511). Written informed consent for participation in the study was obtained before surgery.

### Participants and study settings

Study participants were recruited from March 2009 and September 2009. The inclusion criteria were: (1) primary and elective cardiac surgery; (2) age ≧40 years; (3) with normal liver, kidney and lung preoperative function and without history of diabetes; (4) no history of neurological or psychiatric disorders; (5) ability of patients to communicate verbally and understand the sleep questionnaires administered before surgery and after being transferred from the ICU; (6) length of ICU stay ≥48 hours; (7) Glasgow coma score (GCS) >10 in the first and second postoperative days; and (8) stable hemodynamics postoperatively. Exclusion criteria were: (1) severe sleep disorder requiring daily treatment before surgery; (2) patients with severe postoperative complications; (3) presence of postoperative renal failure; (4) presence of thoracic aortic dissection; (5) postoperative unconsciousness, coma or delirium; and (6) cardiac valve replacement or congenital heart disease requiring sedation and analgesics after surgery. Staff members were asked to continue all usual routines and care practices and to make no special attempts to decrease noise during the study.

### Intervention and randomization

Patients were randomly assigned to two different groups using the closed-envelope method. The control group received routine care during the nights after surgery and the experimental group received protective devices (wearing earplugs and eye masks during nocturnal sleep) with 30 minutes of relaxing music on the basis of routine care.

After randomization, earplugs (3 M Corporation, Beijing, China) and eye masks were provided 2 to 3 days before surgery and patients in the intervention group were asked to wear them. Meanwhile, the researcher explained to them that they should wear the earplugs and eye masks during their postoperative stay in ICU to ensure rest and instructed patients to use them properly. The patients chose from three types of eye mask provided. Providing the earplugs and eye masks preoperatively allowed patients to adapt to wearing earplugs and eye masks, and it also helped to play a role in establishing a time cue. During the postoperative ICU stay, ICU nurses assisted patients with wearing earplugs and eye masks from 9:0 pm every night until the next morning. Pieces of music for relaxing and implying time of day were collected and recorded on an MP3 player. Sounds of nature and bird songs were selected to imply morning. Sounds of frogs and waves were selected to imply evening. Pieces of classical music, including *Blue Danube*, *Morning Song*, *Lofty Mountains and Flowing Water*, *Clouds Chasing the Moon*, *Lotus Emerging out of Water*, and *Moonlight Sonata*, et cetera, were selected as relaxing music. Patients used earphones to listen to the corresponding music at 8:00 to 9:00 pm and 7:30 to 8:30 am every day after surgery. The duration of listening to music was 30 minutes. The music volume was set at a comfortable level for each participant. The MP3 music was supplied through earphones to the participants. Sometimes listening to music had to be stopped due to need for immediate care; when this occurred, the period of listening to music was shifted, although the range remained within the period of 9:00 pm and 8:30 am. During the night when care-givers needed to interact with the patients, whether or not the earplugs and eye masks were retained was left up to the nurses’ judgment, patients’ request and specific circumstances. For patients who did not like the music that was provided, we reselected other pieces of music for them on the basis of the requirement to relax the patients and help them sleep. Those who were strongly disinclined to listen to music were withdrawn from the study. In the control group, no interventions mentioned above were offered to the patients and routine preoperative and postoperative medical care was provided.

Reasons for study termination criteria were: (1) disease aggravation threatening the patient’s life; (2) death; (3) patient request for withdrawal; (4) transfer out of the ICU less than 2 nights postoperatively; and (5) serious adverse reactions.

### Data collection and measures

Demographic and clinical data were collected from the patient’s record. Acute physiology and chronic health evaluation (APACHE) II severity-of-illness scores for the initial 24-hour period of admission to the ICU and preoperative cardiac function were calculated to assess severity of illness.

### Assessment of sleep perception

Subjective sleep quality during the ICU stay was evaluated 1 to 2 days following transfer out of the ICU, using the Chinese version of the Richards-Campbell sleep questionnaire (RCSQ). The original RCSQ had six items and evaluated aspects of nighttime sleep including: (1) depth; (2) latency (time to fall asleep); (3) number of awakenings; (4) efficiency (percent of time awake); (5) quality; and (6) perceived nighttime noise measured on a 100-mm visual-analog scale (VAS) [29]. The RCSQ was pilot-tested in a medical ICU [30] and validated with overnight polysomnography (PSG) in medical ICU patients [29]. Cronbach’s alpha value of the Chinese RCSQ in this study was 0.84; higher scores indicate poorer perceived sleep quality in this Chinese version of the RCSQ. The patients filled out the Pittsburgh sleep quality index questionnaire (PSQI) [31] before surgery to evaluate the quality of sleep one month before surgery.

### Nocturnal melatonin and cortisol levels

Nocturnal urine (12-hour) was collected between 8:00 pm and 8:00 am on the day before surgery and the first and second days after surgery. The containers were wrapped with black plastic to protect the urine from light. The total volume was recorded and two 2-ml samples were frozen to −20°C for later analysis. Concentrations of 6-sulphatoxymelatonin (6-SMT), the stable metabolite of melatonin, were measured by ELISA (IBL, Hamburg, Germany) in duplicate. Concentration of cortisol, a stress-related hormone, was measured in another urine sample by radioimmune assay (RIA) (Beijing North Institute of Biological Technology, Beijing, China).

### Nocturnal noise and light levels

The nocturnal 12-hour (from 8:00 pm to 8:00 am) noise level in the ICU was monitored continuously using a digital sound level meter (model AWA5610, AWAI, Hangzhou, China.) The light intensity between 8:00 pm and 8:00 am in the ICU was measured at the eye level of the patient once every two hours using a light detector (model TES1332,Taiwantes, Shenzen, China).

### Statistical analysis and sample size

Data were analyzed using SPSS version 16.0 (SPSS Inc., Chicago, IL, USA). Measurement data were expressed as mean ± standard deviation and count data were expressed as ratios. The independent samples *t*-test or non-parametric Wilcoxon rank sum test were used for comparison of the groups, and the chi-square (χ^2^) test was used for comparison of count data. One-way repeated measures analysis of variance (ANOVA) was used to determine differences in 6-SMT and cortisol concentrations at different points in time. An alpha of 0.05 was considered significant.

The sample size was calculated based on our pilot study, which found that the estimated standard deviation of mean sleep score in ICU patients was 27. We hypothesized that the non-pharmacological intervention could improve the sleep quality by inducing a 28-point difference of total mean sleep score between groups. Using an effect size of 0.8 and a *P*-value ≤0.05, the required sample size for each group was calculated as 20 per group, but 25 per group were recruited after considering a 10% dropout rate.

## Results

### Sample characteristics

**Figure 1:**
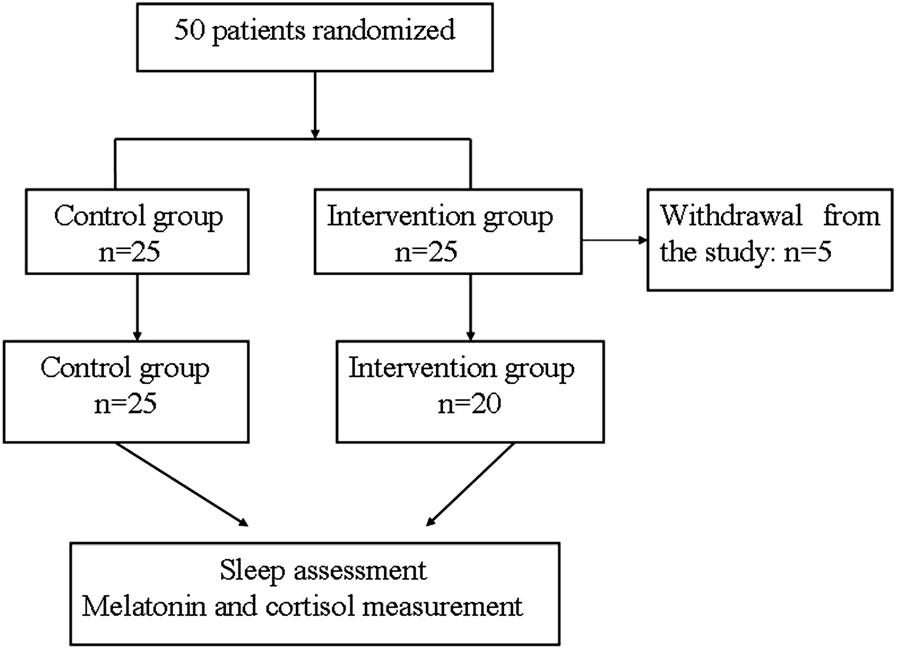
**Flow chart of the study.**

In total 50 patients who met the inclusion criteria were enrolled and randomly divided into the two groups (intervention = 25; control = 25). In the intervention group, five patients were withdrawn due to serious postoperative complications (n = 2), refusal to wear earplugs and eye masks (n = 2), and refusal to listen to music (n = 1). Thus, data analyses were carried out for 20 cases in the intervention group and 25 cases in the control group (Figure 1). The findings of patients’ demographic analysis are shown in Table 1. Both study groups were comparable at baseline, with no significant differences in age, gender, operative time, presence of cardiopulmonary bypass, preoperative cardiac function, APACHE II scores, PSQI scores, duration of mechanical ventilation, length of ICU stay or length of hospital stay (*P* >0.05).

**Table 1:** **Patients demographic characteristics**

**Variable**	**Control group (n = 25)**	**Intervention group (n = 20)**	***P*** **-** ***value***
Age, years, mean ± SD	56.8 ± 11.2	56.6 ± 11	0.97
Gender, number of patients			0.5
Male	16	11	
Female	9	9	
Weight, kg, mean ± SD	61.6 ± 11.7	60.5 ± 12.9	0.6
Surgery, number of patients			
CABG	4	3	0.94
Cardiac value replacement	17	13	
Congenital heart disease	4	4	
Operative time, hours, mean ± SD	3.3 ± 1.2	3.3 ± 0.7	0.96
Cardiopulmonary bypass, number of patients			
Yes	19	14	0.7
No	6	6	
APACHE II scores on admission, mean ± SD	20 ± 3.1	21.2 ± 2.9	0.75
Preoperative cardiac function score, number of patients			
П	3	3	0.6
Ш	21	17	
IV	1	0	
Duration of mechanical ventilation, hours, mean ± SD	22 ± 10.1	22.7 ± 9.5	0.8
Length of ICU stay, hours, mean ± SD	58.9 ± 20	53.0 ± 16	0.29
Length of hospital stay, days, mean ± SD	22.6 ± 10.8	20.7 ± 6.1	0.5
Preoperative PSQI, mean ± SD	7.5 ± 3.7	8.6 ± 4.5	0.3
Discharge outcomes of hospital, number of patients			
No death	23	20	0.4
mortality	2	0	

CABG, coronary artery bypass surgery; PSQI, Pittsburgh sleep quality index; APACHE, acute physiology and chronic health evaluation scoring system.

All of the seven coronary artery bypass graft (CABG) patients used midazolam (0.05 mg/kg/h) plus fentanyl (1 μg/kg/h) for sedative and analgesic during the first 48 hours post surgery.

### Perception of sleep quality

The independent samples *t*-test showed subjective sleep quality in the intervention group was significantly higher than in the control group (*P* <0.05). Significant differences were also found between groups in the five items of sleep scoring. Patients’ perceptions of nighttime noise were significantly lower in the experimental group than in the control group (*P* <0.05) (Table 2).

**Table 2:** **Comparison of sleep scores between groups**

**Variables, mean ± SD**	**Intervention group**	**Control group**	***P*** **-** ***value***
Depth	26.7 ± 21.5	55.5 ± 27.4	0.00
Latency (time to fall asleep)	23.7 ± 17.4	60.4 ± 25.9	0.00
Number of awakenings	25.3 ± 16.2	51.2 ± 26.7	0.00
Efficiency (percent of time awake)	21.7 ± 20.9	63.4 ± 21.9	0.00
Perceived quality	23.7 ± 20.6	54.0 ± 25.5	0.00
Perceived nighttime noise	25.0 ± 24.0	40.2 ± 28.8	0.047

### Nocturnal urinary excretion of 6-SMT and cortisol

**Figure 2:**
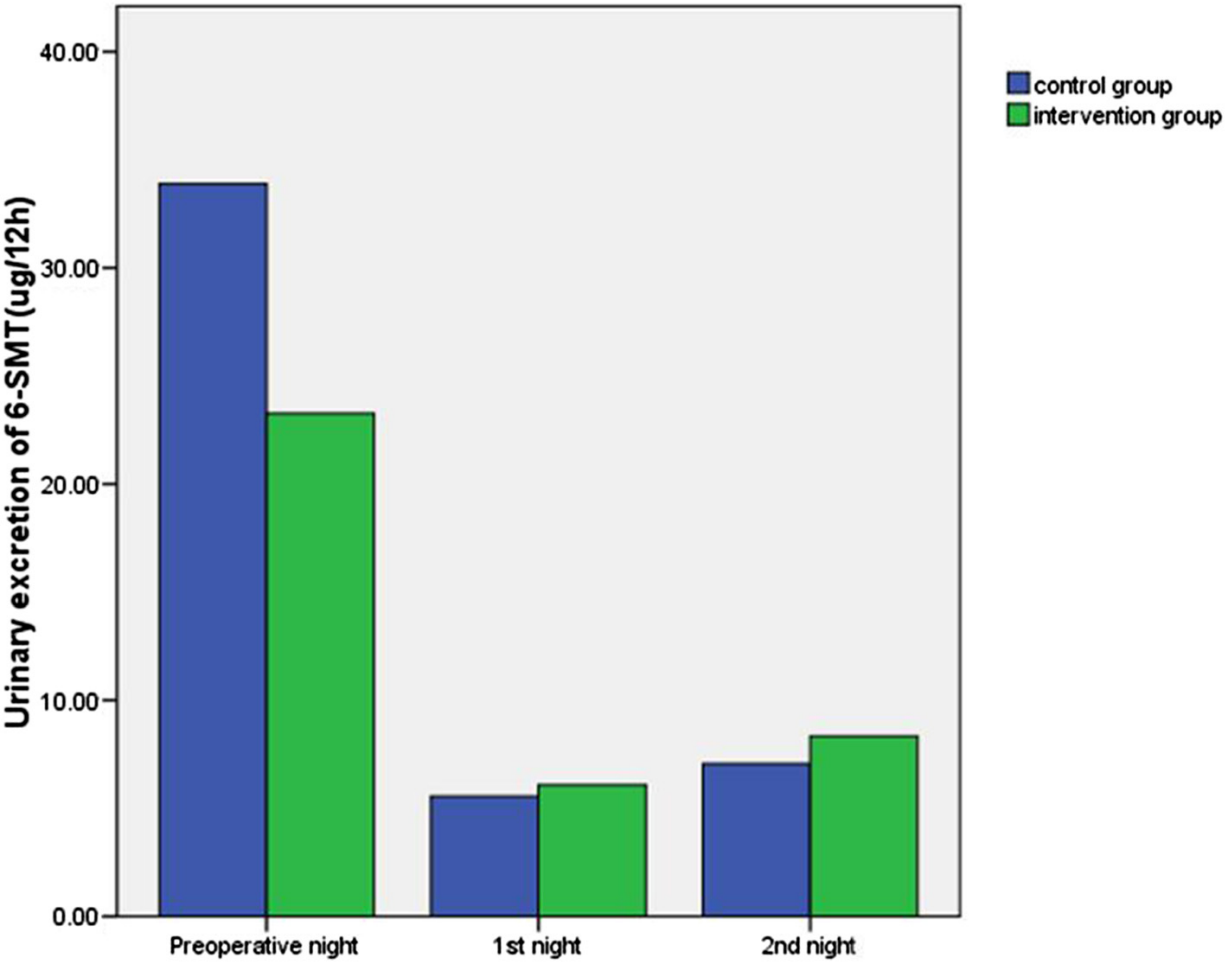
**Comparison between groups of urinary excretion of 6-SMT during the night before surgery, and the first and second postoperative nights.** No significant differences were found between the two groups in 6-SMT levels during the night before surgery, or the first and second postoperative nights (*P* >0.05). 6-SMT, 6-sulphatoxymelatonin.

**Figure 3:**
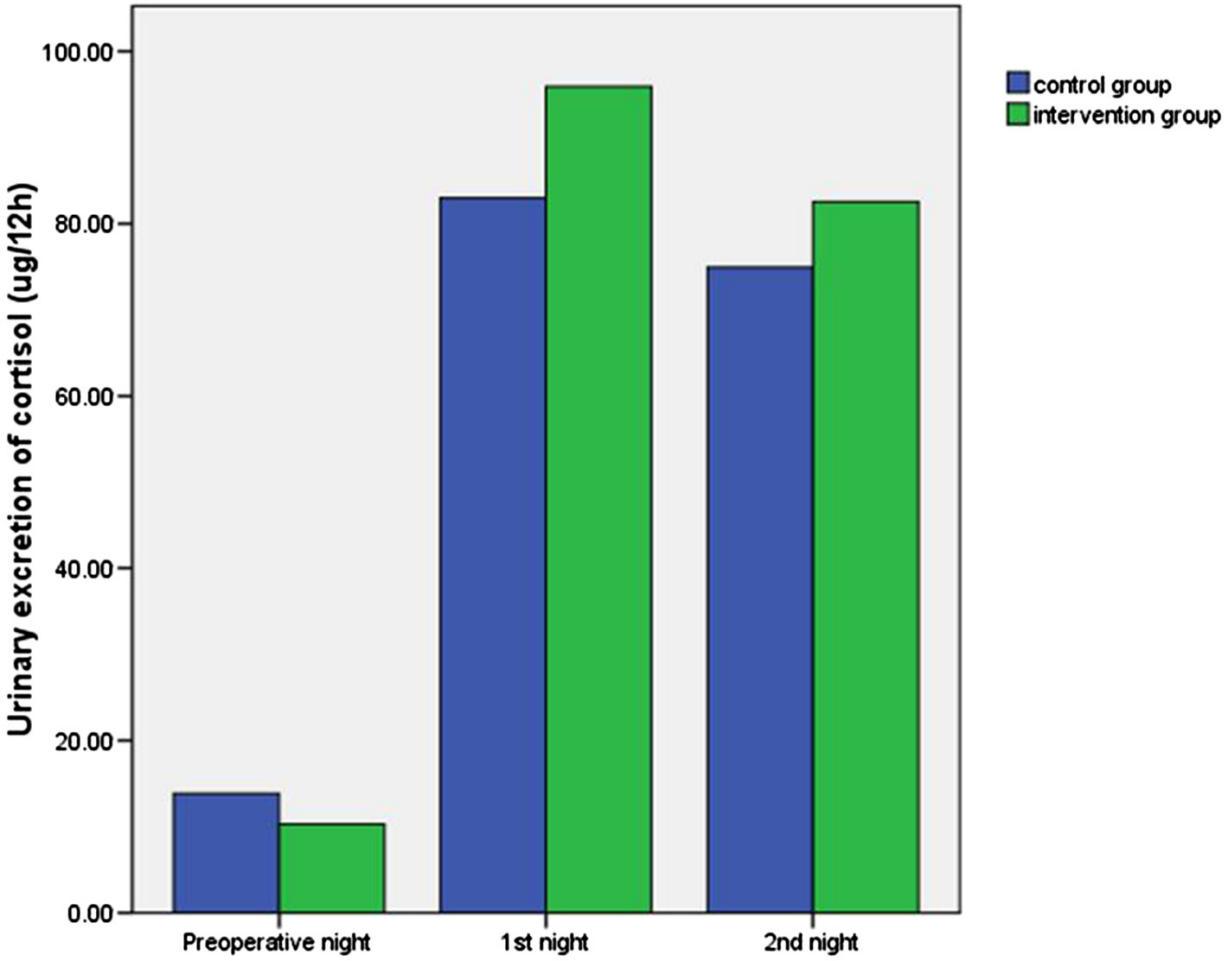
**Urinary excretion of cortisol of the night before surgery, the 1st and 2nd postoperative nights between groups.** No significant differences were found in cortisol levels between the two groups during the night before surgery, and the first and second postoperative nights (*P* >0.05).

Total 12-hour urinary excretion of 6-SMT and cortisol (8:00 pm to 8:00 am) in patients during the night before surgery, and the first and second postoperative nights are shown in Figures 2 and 3, respectively. The Wilcoxon rank sum test showed no significant differences were found between the two groups in 6-SMT levels during the night before surgery (*Z* = −1.27, *P* = 0.22), or the first (*Z* = −0.52, *P* = 0.61) and second postoperative nights (*Z* = −0.03, *P* = 0.97). There were also no significant differences in cortisol levels between the two groups during the night before surgery (*t* = 0.99, *P* = 0.33), or the first (*t* = −0.64, *P* = 0.53) and second postoperative nights (*t* = −0.45, *P* = 0.65) (Table 3).

**Table 3:** **Urinary melatonin and cortisol levels in the groups during the night before surgery, and the first and second nights after surgery**

**Variables**	**Intervention group**				**Control group**			
	**Pre-surgery night**	**First night post surgery**	**Second night post surgery**	***P*** **-value**	**Pre-surgery night**	**First night post surgery**	**Second night post surgery**	***P*** **-value**
6-SMT, ug	23.3 ± 24.3^a^	6.1 ± 9.9^a^	8.3 ± 12.6^a^	0.01	33.9 ± 99.9^a^	5.6 ± 12.7^a^	7.1 ± 9.8^a^	0.00
Cortisol, ug	10.3 ± 8.3^b^	95.9 ± 71.2^b^	82.5 ± 47.3^b^	0.00	13.8 ± 8.8^b^	82.9 ± 56.9^b^	74.9 ± 56.3^b^	0.00

^a^No significant differences were found between the two groups in 6-SMT levels during the night before surgery (*P* = 0.22), or the first (*P* = 0.61) and second postoperative nights (*P* = 0.97). ^b^No significant differences were found in cortisol levels between the two groups during the night before surgery (*P* = 0.33), or the first (*P* = 0.53) and second postoperative nights (*P* = 0.65).

Repeated measures ANOVA showed the 6-SMT levels of the first and second postoperative nights were significantly lower than those of the night before surgery (*F* = 7.53, *P* = 0.01). The cortisol levels of the first and second postoperative nights were significantly higher than those of the night before surgery (*F* = 88.63, *P* = 0.00).

### Nocturnal noise and light levels

No significant differences between the groups were observed in the mean noise level during nighttime (intervention: 69.8 ± 2 dB(A); controls: 69.6 ± 2.2 dB(A)). There were no significant differences between groups in nighttime noise (*P* = 0.6). Mean light level during nighttime also did not differ (intervention: 167.1 ± 5 lux; control: 170.2 ± 8 lux).

## Discussion

Adequate sleep is a required condition for recovery after serious illness. Previous studies have reported that patients suffer severe sleep disturbances after cardiac surgery [32,33]. It is essential in clinical practice to control or attenuate various factors disrupting sleep and thus, maximize patients’ ability to experience restorative sleep. Overall, these results support the notion that using protective devices (earplugs and eye masks) plus listening to sleep-music during the night can significantly improve subjective sleep quality in an ICU setting.

We found that the mean preoperative PSQI scores of the two groups were more than seven points in this study, indicating that the preoperative sleep quality of the participants in both groups were generally poor. Difficulty in falling asleep and difficulty in staying asleep were the two main symptoms, similar to data reported by Redeker [32].

It has been reported that ICU patients are exposed to an environment with high noise levels and continuous day-to-night lighting [4,8,13-15]. Continuous monitoring and care are commonly needed in the ICU and patients find themselves surrounded by medical and technological equipment. Aside from their presenting health problem, its treatment and care, the ICU environment may increase stress among patients [34]. Our previous study indicated that using earplugs and eye masks can improve REM sleep and sleep quality in healthy subjects in a simulated ICU environment [28]. The results are similar to those of other studies using earplugs and eye masks [24,35,36].

Patients’ compliance with and tolerability of these interventions is critical. It has been reported that some ICU patients refuse to wear earplugs and eye masks because they feel uncomfortable, cannot see anything or feel pain after wearing them [35,37]. We found similar responses in three patients in this study. One patient described nervousness, a feeling of panic and a sense of suffocation after wearing an eye mask and earplugs and another reported feeling pain in the ear canal with the ear plugs. One patient withdrew after listening to music for only 5 minutes. The objective of protective intervention and music therapy is to help patients fall asleep and maintain sleep by reducing interference from potentially noxious environmental stimuli and relieving their anxiety with soothing music. Therefore, the prerequisite for applying a certain method must be that the patient readily accepts this kind of method. This suggests that the ICU staff must actively help patients to understand the benefits before applying the intervention. The nurses should assess individual variability in sensitivity or anatomy of the ears and patients’ acceptability of the protective devices prior to using them. Medical staff members should learn how to apply earplugs and eye masks properly to help patients benefit. For example, critical care nurses should help patients select the appropriate size of earplugs and eye masks, and provide accurate instructions and assistance for their use, especially for how to insert earplugs properly, and minimize any transient discomfort.

Melatonin is a major regulator of circadian rhythm in humans, which differs from evidence for circadian rhythm that melatonin is not secreted in specific strains of mice [38]. Melatonin is secreted from the pineal gland, while cortisol is one of the major glucocorticoid hormones secreted by the adrenal cortex. Both play a role in the regulation of the sleep-wake cycle. Melatonin secretion follows the day-night cycle, with levels normally low during daytime, increasing soon after onset of darkness, and peaking in the middle of the night [39]. Cortisol levels tend to run in an opposite pattern, with peak levels occurring 30 minutes after awakening [40]. Several studies have suggested that sleep disorders and cognitive dysfunction in ICU patients may be associated with disruption of melatonin secretion [41,42]. Persistent high cortisol levels may affect metabolism, organ function, and immune function. These physiological sequelae are not conducive to recovery. In the present study no significant differences were found in 6-SMT levels and cortisol levels between the two groups during the three nights, yet the level of nocturnal melatonin secretion decreased significantly, while the level of cortisol secretion increased significantly on both postoperative nights. Previous studies indicated that medication (such as analgesics, sedative, and beta-blockers), surgery and anesthesia may influence the secretion of melatonin and cortisol [43,44]. Therefore, all of these factors may play a role in the melatonin and cortisol results and mask potential effects of the intervention in our study.

Our levels of 6-SMT were found to be lower than those reported in a previous study of 40 patients in a surgical ICU [45]. There is great interpatient variability in absolute 6-SMT levels; indeed, we observed 20-fold inter-individual variability in 6-SMT levels [45-48] in our patient population. Thus, large sample sizes are required to observe significant between-group differences. Melatonin secretion varies with age and to some extent with gender [49]. Varying urine collection strategies also make it difficult to make cross-study comparisons.

Environmental light is a main *zeitgeber* of the circadian rhythms, and can affect melatonin secretion [46]. Environmental factors such as ambient noise are also main synchronizers [50]. ICU usually requires continuous artificial lighting at night. Although we tried to reduce the influence of light and noise disturbance on patients by offering them earplugs and eye masks at night, and provided music therapy to relax and imply time of day and help patients sleep, the results still showed that the effort did not significantly impact biological measures related to light-dark transitions. Our ICU has very few windows so artificial lighting is required for daytime lighting, which can result in loss of patients’ accurate cognition of time and space, inducing disruption in patients’ biological clocks and affecting sleep quality. The suprachiasmatic nucleus (SCN), the central circadian pacemaker in mammalians, can be altered by cognition [51]. Our results are not entirely consistent with the results of our previous study in the sleep laboratory [28]. Compared with healthy subjects, ICU patients’ sleep, melatonin and cortisol secretion are not only affected by noise and light, but likely also by many other factors, including their disease, admission to an ICU, surgical intervention and medications, which may all contribute to the differences in results between the previous simulated experiment and the present clinical trial.

### Limitations of the study and suggestions for future studies

Our study design has a number of limitations, which should be noted. First, the study only evaluated subjective sleep quality and did not carry out an objective sleep assessment. PSG is the gold-standard of sleep measurement. However, PSG application is limited in the ICU due to its high cost and inconvenient manipulation. Second, the study only assessed a 12-hour nocturnal period rather than over 24 hours during the first two nights in ICU. ICU patients experience circadian rhythm disturbances with sleep traversing day and night. Therefore, an ideal study should measure the sleep pattern and circadian rhythm over multiple 24-hour periods. Moreover, this study included a specific population in a CSICU. Therefore, results may not be applicable to all settings and all patients. In addition, our sample sizes were small, which limited the power of our statistical analyses. Future studies with larger and more diversity of the participants would likely support these recommendations.

## Conclusions

In summary, our results clearly demonstrated the combination of using earplugs and eye masks with relaxing background music is useful for promoting sleep in CSICU adult patients, but the underlying mechanisms are more complex than simple changes in levels of 6-SMT and cortisol. Our pilot study provides a reasonable basis for promoting these non-pharmacological interventions for ICU patients. Future study designs to replicate our results should consider including larger samples, include more diverse ICU populations, extend the time frame for data collection and post-discharge follow up to determine any longer-term benefits of this intervention.

## Key messages

- Using earplugs and eye masks with relaxing background music is useful for promoting the sleep perception of the patient
- Using earplugs and eye masks with relaxing background music does not influence the nocturnal melatonin or cortisol levels

## Abbreviations

(A): weighting is relating to the measurement of sound pressure level
6-SMT: 6-sulphatoxymelatonin
ANOVA: one-way repeated measures analysis of variance
APACHE: acute physiology and chronic health evaluation scoring system
CABG: coronary artery bypass surgery
CSICU: Cardiac surgical intensive care unit
ELISA: enzyme-linked immunosorbent assay
GCS: Glasgow coma score
PSG: polysomnography
PSQI: Pittsburgh sleep quality index
RCSQ: Richards-Campbell sleep questionnaire
RCT: randomized controlled trial
REM: rapid eye movement
WHO: World Health Organization

## References

[1] Parthasarathy S, Tobin MJ (2002). Effect of ventilator mode on sleep quality in critically ill patients. Am J Respir Crit Care Med.

[2] Richardson A, Crow W, Coghill E, Turnock C (2007). A comparison of sleep assessment tools by nurses and patients in critical care. J Clin Nurs.

[3] Whitcomb JJ, Morgan M, Irvin T, Spencer K, Boynton L, Turman S (2013). A pilot study on delirium in the intensive care unit: a creative inquiry project with undergraduate nursing students. Dimens Crit Care Nurs.

[4] Mejer TJ, Eveloff SE, Bauer MS, Schwartz WA, Hill NS, Millman RP (1994). Adverse environmental conditions in the respiratory and medical ICU settings. Chest.

[5] Weinhouse GL, Schwab RJ, Watson PL, Patil N, Vaccaro B, Pandharipande P (2009). Bench-to-bedside review: delirium in ICU patients - importance of sleep deprivation. Crit Care.

[6] Kamdar BB, Needham DM, Collop NA (2012). Sleep deprivation in critical illness: its role in physical and psychological recovery. J Intensive Care Med.

[7] Cooper AB, Thornley KS, Young GB, Slutsky AS, Stewart TE, Hanly PJ (2000). Sleep in critically ill patients requiring mechanical ventilation. Chest.

[8] Freedman NS, Gazendam J, Levan L, Pack AI, Schwab RJ (2001). Abnormal sleep/wake cycles and the effect of environmental noise on sleep disruption in the intensive care unit. Am J Respir Crit Care Med.

[9] Friese RS, Diaz-Arrastia R, McBride D, Frankel H, Gentilello LM (2007). Quantity and quality of sleep in the surgical intensive care unit: are our patients sleeping?. J Trauma.

[10] Freedman NS, Kotzer N, Schwab RJ (1999). Patient perception of sleep quality and etiology of sleep disruption in the intensive care unit. Am J Respir Crit Care Med.

[11] Andersen JH, Boesen HC, Skovgaard OK (2013). Sleep in the Intensive Care Unit measured by polysomnography. Minerva Anestesiol.

[12] Tembo AC, Parker V (2009). Factors that impact on sleep in intensive care patients. Intensive Crit Care Nurs.

[13] Aaron JN, Carlisle CC, Carskadon MA, Meyer TJ, Hill NS, Millman RP (1996). Environmental noise as a cause of sleep disruption in an intermediate respiratory care unit. Sleep.

[14] Drouot X, Cabello B, d’Ortho M, Brochard L (2008). Sleep in the intensive care unit. Sleep Med Rev.

[15] Walder B, Francioli D, Meyer JJ, Lançon M, Romand JA (2000). Effects of guidelines implementation in a surgical intensive care unit to control nighttime light and noise levels. Crit Care Med.

[16] Kass JL (2008). To sleep in an intensive care unit, perchance or heal. Crit Care Med.

[17] Berglund B, Lindvall T, Schwela DH. Guidelines for Community Noise Geneva: World Health Organization. 1999. http://whqlibdoc.who.int/hq/1999/a68672.pdf.

[18] Yu HZ, Ma AY, Huang SC (2003). The effect of noise on the circulatory function and heart rate variability in workers. J Environ Occup Med.

[19] Chang AM, Aeschbach D, Duffy JF, Czeisler CA (2015). Evening use of light-emitting eReaders negatively affects sleep, circadian timing, and next-morning alertness. Proc Natl Acad Sci U S A.

[20] Chellappa SL, Steiner R, Oelhafen P, Lang D, Götz T, Krebs J (2013). Acute exposure to evening blue-enriched light impacts on human sleep. J Sleep Res.

[21] Chen JH, Chao YH, Lu SF, Shi TF, Chao YF (2012). The effectiveness of valerian acupressure on the sleep of ICU patients: a randomized clinical trial. Int J Nurs Stud.

[22] Richards KC (1998). Effect of a back massage and relaxation intervention on sleep in critically ill patients. Am J Crit Care.

[23] Jaber S, Bahloul H, Guétin S, Chanques G, Sebbane M, Eledjam J (2007). Effects of music therapy in intensive care unit without sedation in weaning patients versus non-ventilated patients. Ann Fr Anesth Reanim.

[24] Le Guen M, Nicolas-Robin A, Lebard C, Arnulf I, Langeron O (2014). Earplugs and eye masks vs routine care prevent sleep impairment in post-anaesthesia care unit: a randomized study. Br J Anaesth.

[25] Van Rompaey B, Elseviers MM, Van Drom W, Fromont V, Jorens PG. The effect of earplugs during the night on the onset of delirium and sleep perception: a randomized controlled trial in intensive care patients. Crit Care. 2012;3:16:R73. 10.1186/cc11330 PMC3580615 22559080

[26] Wallace CJ, Robins J, Alvord LS, Walker JM (1999). The effects of earplugs on sleep measures during exposure to simulated intensive care unit noise. Am J Crit Care.

[27] Topf M, Davis JE (1993). Critical care unit noise and rapid eye movement (REM) sleep. Heart Lung.

[28] Hu RF, Jiang XY, Chen XY, Zhang YH (2010). Effects of earplugs and eye masks on nocturnal sleep, melatonin and cortisol in a simulated intensive care unit environment. Crit Care.

[29] Richards KC, O’Sullivan PS, Phillips RL (2000). Measurement of sleep in critically ill patients. J Nurs Meas.

[30] Richards KC, Bairnsfather L (1988). A description of night sleep patterns in the critical care unit. Heart Lung.

[31] Liu XC. Rating scales for mental health. In: Wang XD, editor. Chinese Mental Health Journal, 1999; 375–78

[32] Redeker NS, Ruggiero J, Hedges C (2004). Patterns and predictors of sleep pattern disturbance after cardiac surgery. Res Nurs Health.

[33] Edell-Gustafsson UM, Hetta JE, Aren GB, Hamrin EK (1997). Measurement of sleep and quality of life before and after coronary artery bypass grafting: a pilot study. Int J Nurs Pract.

[34] Engwall M, Fridb I, Bergbom I, Lindabl B (2014). Let there be light and darkness. Crit Care Nurs Q.

[35] Richardson A, Allsop M, Coghill E, Turnock C (2007). Earplugs and eye masks: do they improve critical care patients’ sleep?. Nurs Crit Care.

[36] Koo YJ, Koh HJ (2008). Effects of eye protective device and ear protective device application on sleep disorder with coronary disease patients in CCU. J Korean Acad Nurs.

[37] Scotto C, McClusky C, Spillan S, Kimmel J (2009). Earplugs improve patients’ subjective experience of sleep in critical care. Nurs In Crit Care.

[38] Goto M, Oshima I, Tomita T, Ebihara S (1989). Melatonin content of the pineal gland in different mouse strains. J Pineal Res.

[39] Weitzman ED, Weinberg U, D’ Eletto R, Lynch H, Wurtman RJ, Czeisler C (1978). Studies of the 24 hour rhythm of melatonin in man. J Neural Transm Suppl.

[40] Stone AA, Schwartz JE, Smyth J, Kirschbaum C, Cohen S, Hellhammer D (2001). Individual differences in the diurnal cycle of salivary free cortisol: a replication of flattened cycles for some individuals. Psychoneuroendocrinology.

[41] Shilo L, Dagan Y, Smorjk Y, Weinberg U, Dolev S, Komptel B (1999). Patients in the intensive care unit suffer from severe lack of sleep associated with loss of normal melatonin secretion pattern. Am J Med Sci.

[42] Yin YQ, Luo AL, Guao XY, Li LH, Ren HZ, Ye TH (2005). Changes in perioperative plasma melatonin, cortisol and neuron-specific enolase and neuropsychological function in patients who develop CNS complication after coronary artery bypass. Ch J Anesth.

[43] Kärkelä J, Vakkuri O, Kaukinen S, Huang WQ, Pasanen M (2002). The influence of anaesthesia and surgery on the circadian rhythm of melatonin. Acta Anaesthesiol Scand.

[44] Govitrapong P, Pariyanonth M, Ebadi M (1992). The presence and actions of opioid receptors in bovine pineal gland. J Pineal Res.

[45] Riutta A, Pauli Y, Kaukinen S (2009). Diurnal variation of melatonin and cortisol is maintained in non-septic intensive care patients. Intensive Care Med.

[46] Skene DJ, Arendt J (2006). Human circadian rhythms: physiological and therapeutic relevance of light and melatonin. Ann Clin Biochem.

[47] Benloucif S, Burgess HJ, Klerman EB, Lewy AJ, Middleton B, Murphy PJ (2008). Measuring melatonin in humans. J Clin Sleep Med.

[48] Mahlberg R, Tilmann A, Salewski L, Kunz D (2006). Normative data on the daily profile of urinary 6-sulfatoxymelatonin in healthy subjects between the ages of 20 and 84. Psychoneuroendocrinology.

[49] Wetterberg L, Bratlid T, von Knorring L, Eberhard G, Yuwiler A (1999). A multinational study of the relationships between nighttime urinary melatonin production, age, gender, body size, and latitude. Eur Arch Psychiatry Clin Neurosci.

[50] Wang ZR (2006). Chronobiology.

[51] Gritton HJ, Stasiak AM, Sarter M, Lee TM (2013). Cognitive performance as a Zeitgeber: cognitive oscillators and cholinergic modulation of the SCN entrain circadian rhythms. PLoS One.

